# Supplementary figures and images for: Comprehensive transcriptomic analysis of long non-coding RNAs in bovine ovarian follicles and early embryos
Source: PLoS One. 2023 Sep 19;18(9):e0291761. doi: 10.1371/journal.pone.0291761 (PMC10508637; doi:10.1371/journal.pone.0291761)

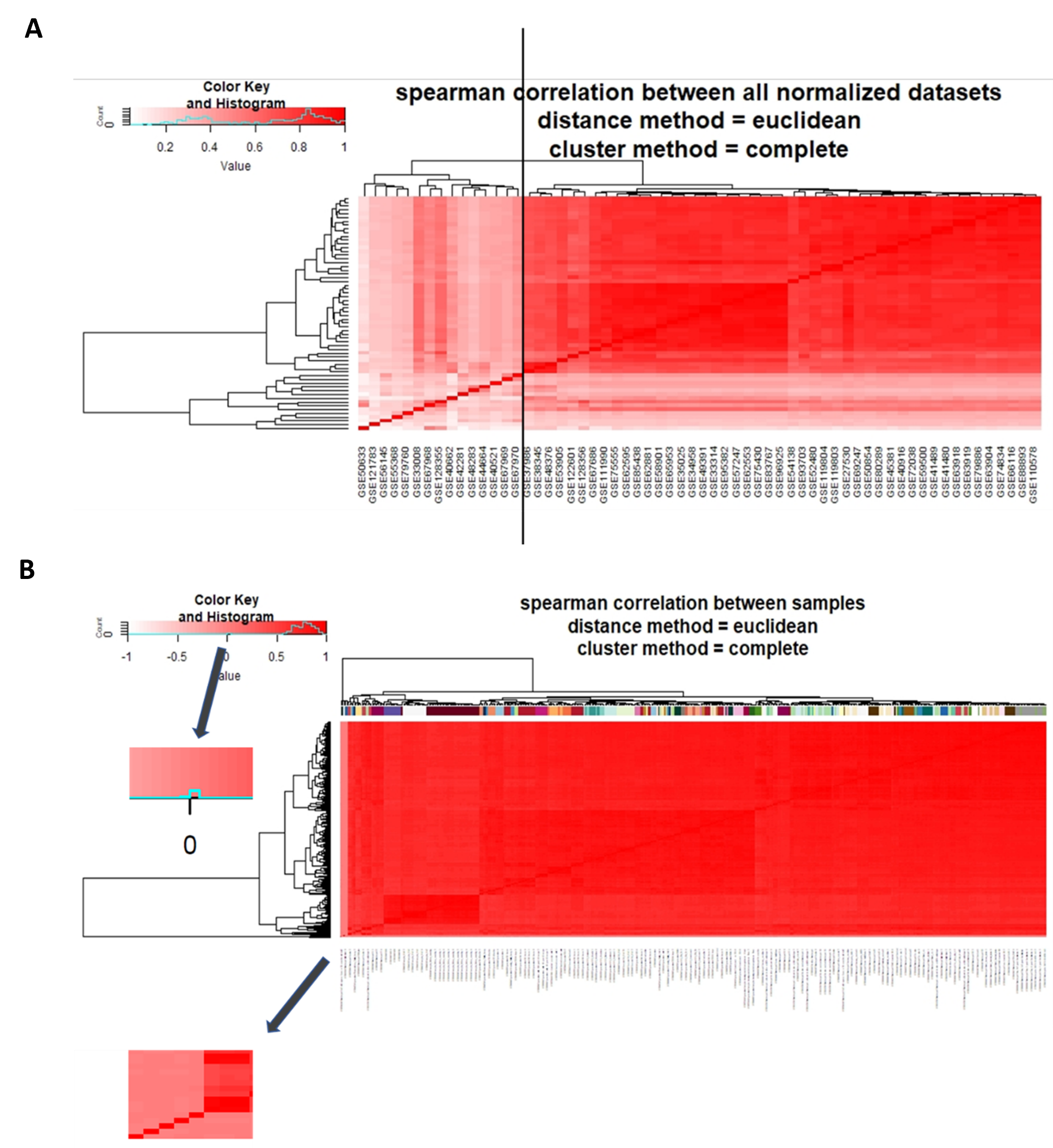

Supplement: S1 Fig — (A) Correlation analysis across datasets (N = 62). (B) Correlation analysis across samples (N = 473). The arrow represents outlier samples. (TIF) [file pone.0291761.s001.tif]

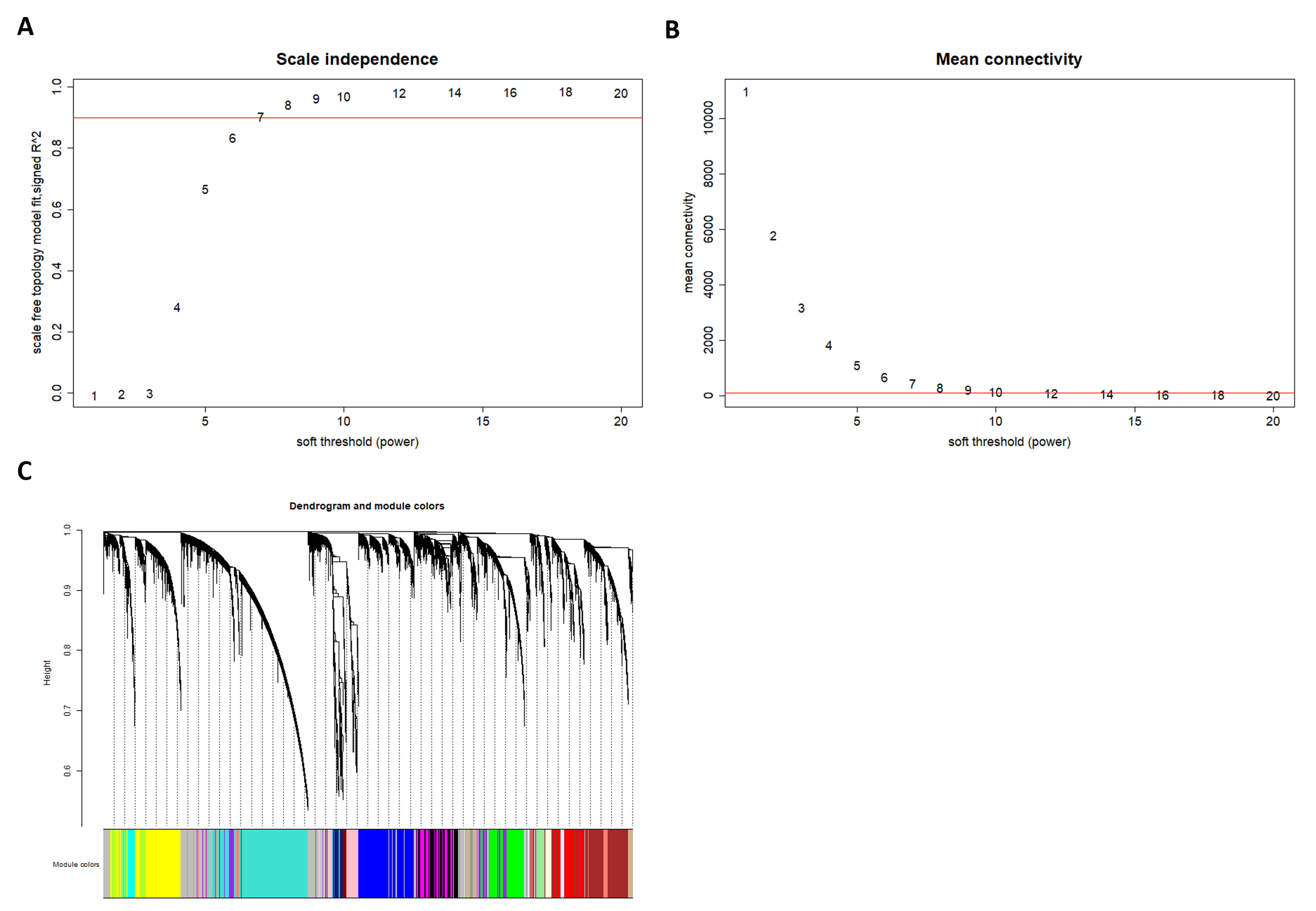

Supplement: S2 Fig — (A) Scale-free topology model fit for determining the optimal soft threshold. (B) mean connectivity analysis for selecting the optimal soft threshold. (C) The cluster dendrogram and module colors of 21809 transcripts. (TIF) [file pone.0291761.s002.tif]

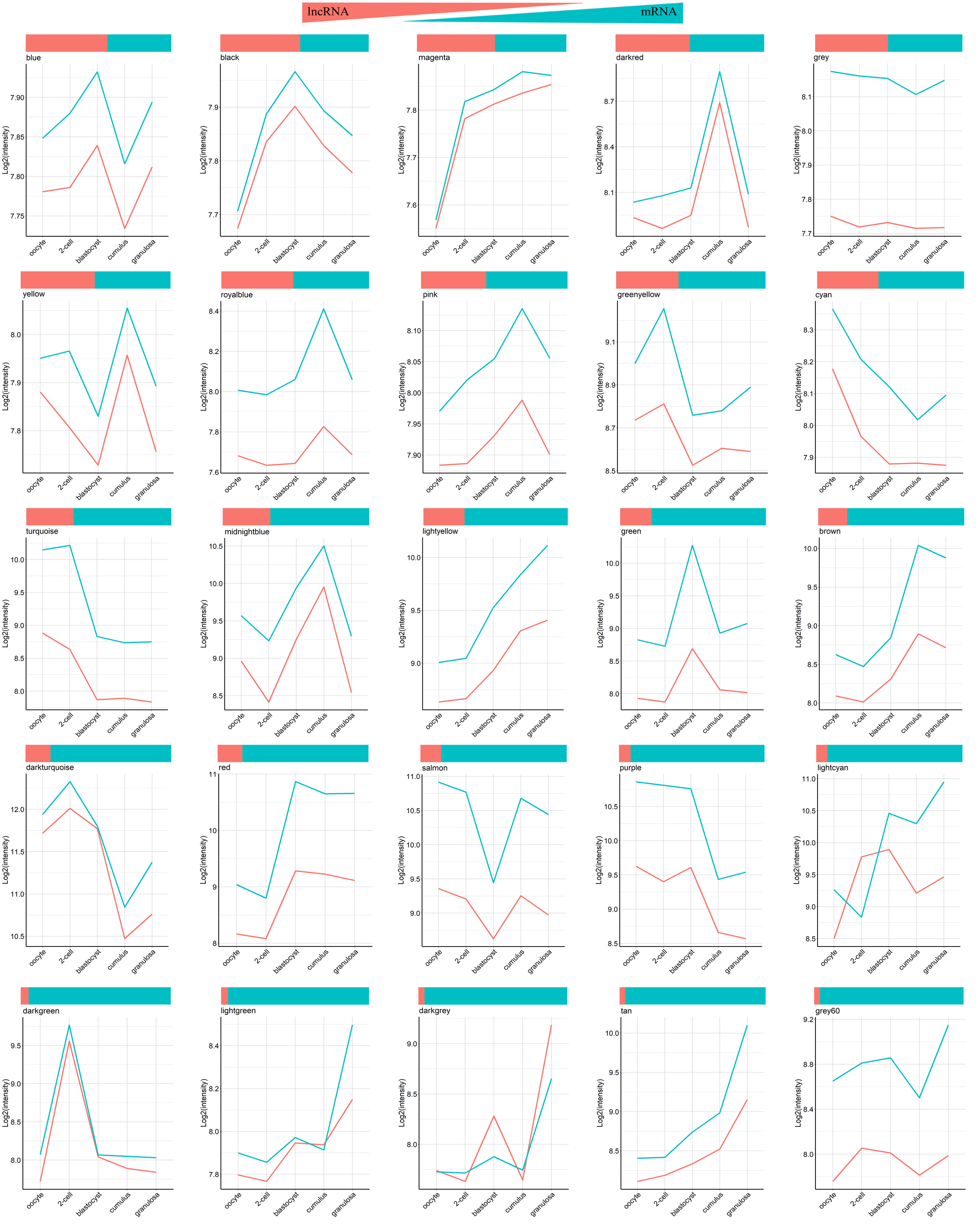

Supplement: S3 Fig — The lines represent the mean expression levels. Modules are arranged by decreasing fraction of lncRNAs. The bars above each graph show the proportion of lncRNAs and mRNAs in each module. (TIF) [file pone.0291761.s003.tif]

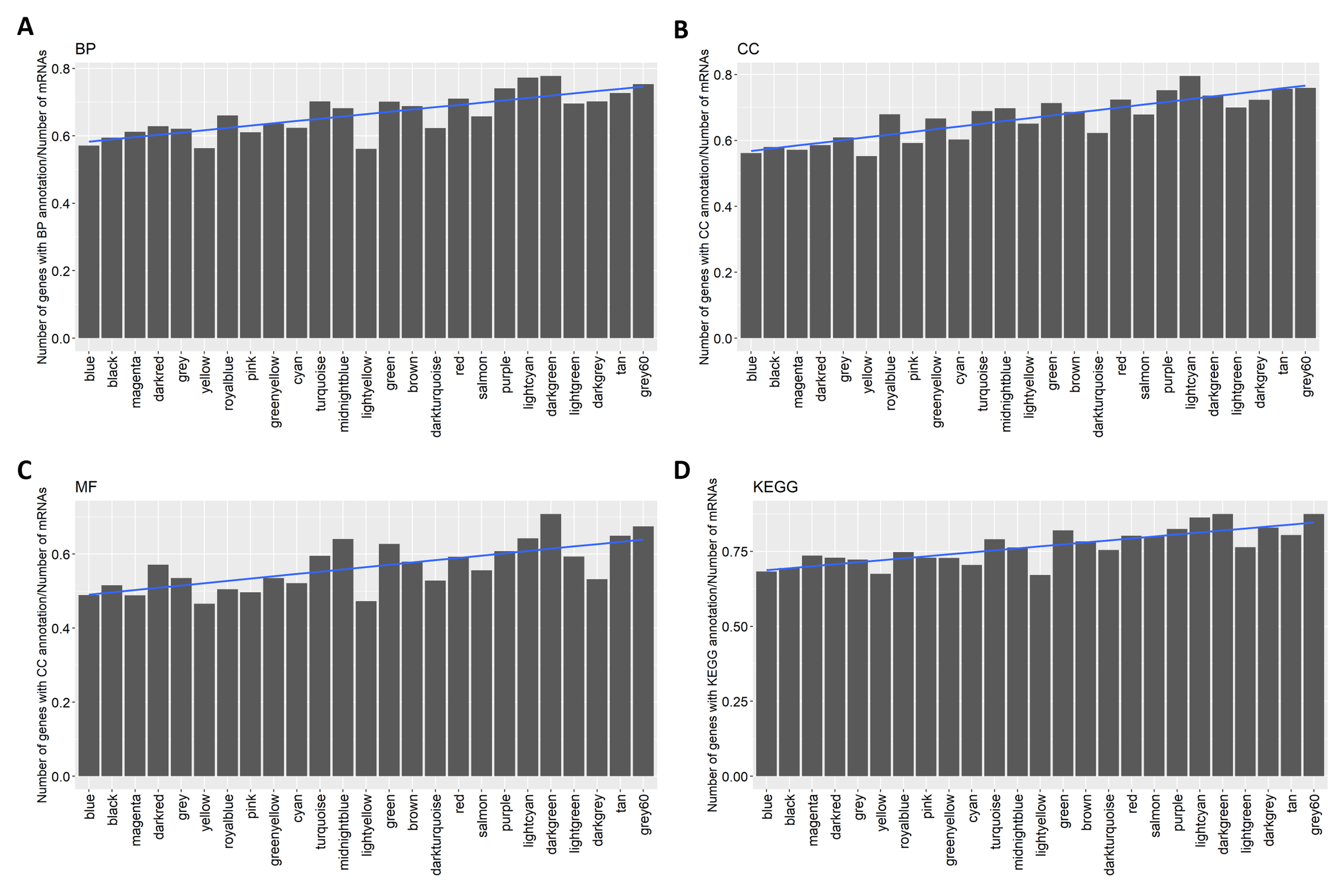

Supplement: S4 Fig — (A) the fraction of mRNAs with BP annotations. (B) the fraction of mRNAs with CC annotations. (C) the fraction of mRNAs with MF annotations. (D) the fraction of mRNAs with KEGG annotations. The blue line represents the linear regression. Abbreviations: BP, biological process; CC, cellular component; MF, molecular function; KEGG, Kyoto Encyclopedia of Genes and Genomes. (TIF) [file pone.0291761.s004.tif]
