# Supplementary material for: Comprehensive transcriptomic analysis of long non-coding RNAs in bovine ovarian follicles and early embryos
Source: PLoS One. 2023 Sep 19;18(9):e0291761. doi: 10.1371/journal.pone.0291761 (PMC10508637; doi:10.1371/journal.pone.0291761)
Supplement: S1 Table — (DOCX) [file pone.0291761.s005.docx]

**S1 Table. Definition and number of probes for different types.**

| **Probes** | **Definition** | **Number** |
| --- | --- | --- |
| Total number of probes |  | 21,809 |
| mRNA | Overlap with exons of protein-coding genes on the same strand | 14,085 |
| lncRNA |  | 7,724 |
| Genic lncRNA | Overlapping protein-coding genes | 2,452 |
| Intronic lncRNA (lincRNA) | Within introns of protein-coding genes on the same strand | 958 |
| Antisense lncRNA (lncNAT) | Overlap with protein-coding genes on the opposite strand | 1,524 (30 overlap with lincRNA) |
| Intergenic lncRNA  (lincRNA) | Not overlapping with protein-coding genes | 5,272 |
| Isolated | >50 kb of protein-coding genes ignoring strand | 1,120 |
| Same strand | >1 kb and <=50 kb of protein-coding genes and transcribed from the same strand | 1,688 |
| Opposite strand | >1 kb and <=50 kb of protein-coding genes and transcribed from the opposite strand | 1,523 |
| UTR-Related lincRNA | Within 1 kb of protein-coding genes on the same strand | 501 |
| UTR-Related antisense lincRNA | Within 1 kb of protein-coding genes on the opposite strand | 440 |
